# Vitamin E therapy prevents the accumulation of congophilic amyloid plaques and neurofibrillary tangles in the hippocampus in a rat model of Alzheimer’s disease

**DOI:** 10.22038/IJBMS.2019.38165.9067

**Published:** 2020-01

**Authors:** Mehrdad Jahanshahi, Emsehgol Nikmahzar, Ali Sayyahi

**Affiliations:** 1 Neuroscience Research Center, Department of Anatomy, Faculty of Medicine, Golestan University of Medical Sciences, Gorgan, Iran; 2 Neuroscience Research Center, Golestan University of Medical Sciences, Gorgan, Iran

**Keywords:** Amyloid plaque, Hippocampus, Neurofibrillary tangles, Rat, Vitamin E

## Abstract

**Objective(s):**

Vitamin E may have beneficial effects on oxidative stress and Aβ-associated reactive oxygen species production in Alzheimer’s disease. But, the exact role of vitamin E as a treatment for Alzheimer’s disease pathogenesis still needs to be studied. Hence, we examined the therapeutic effects of vitamin E on

the density of congophilic amyloid plaques and neurofibrillary tangles in rats’ hippocampi.

**Materials and Methods:**

Wistar rats were randomly assigned to control (no drug treatment), sham scopolamine (3 mg/kg)+saline and Sham scopolamine+sesame oil groups, and three experimental groups that received scopolamine+vitamin E (25, 50, and 100 mg/kg/day) daily for 14 days after scopolamine injection. The rats’ brains were collected immediately following transcardial perfusion and fixed in 4% paraformaldehyde. Pathological brain alterations were monitored through Congo red and bielschowsky silver staining.

**Results:**

Scopolamine treatment led to a significant increase in the density of congophilic amyloid plaques and neurofibrillary tangles in the hippocampus. IP injection of vitamin E in three doses (25, 50, and 100 mg/kg/day) significantly reversed the scopolamine-induced increase of the congophilic amyloid plaque density and density of neurofibrillary tangles in the hippocampus. Although vitamin E (25 and 50 mg/kg/day) doses were also effective, but a 100 mg/kg/day dose of vitamin E was more effective in the reduction of congophilic amyloid plaque and neurofibrillary tangle density.

**Conclusion:**

Vitamin E could exert a therapeutic effect in the reduction of congophilic amyloid plaque and neurofibrillary tangle density in the hippocampus of scopolamine-treated rats and it is useful for Alzheimer’s disease.

## Introduction

The important signs of Alzheimer’s disease (AD) include the accumulation of β-amyloid (Aβ) plaques outside neurons and neurofibrillary tangles inside neurons (1, 2), in endangered brain regions, in particular the hippocampus and cortex (3, 4). Indeed, these histopathologic injuries limit selective brain regions involved in memory and cognitive function (5, 6).

Scopolamine, a muscarinic antagonist, crosses the blood-brain barrier and because of a cholinergic signaling blockade, it was used as a cognitive impairment model associated with AD (7-9). Scopolamine also can induce Aβ accumulation and tau hyperphosphorylation, which are the two neuropathological symptoms of AD, in the rodent brain (10-12).

Tau protein is produced by neurons and found in the cell body and axons. This protein has a major role in the microtubule assembly and its stability maintenance (6). In AD, tau kinases activate by Aβ, which forms a neurofibrillary tangle by hyperphosphorylation of tau protein (13). The hyperphosphorylated form of tau protein causes neurotoxic effects in this disease (6), including mitochondrial impairment and induction of oxidative stress (14). Hyperphosphorylated tau protein accumulates in somatodendritic parts of the neurons and becomes the core component of neurofibrillary tangles. In the early stages of AD, neurofibrillary tangles appear in the entorhinal cortex and with disease progression, these tangles extend to the hippocampus and cortex (1). The number of neurofibrillary tangles is associated with the severity of dementia in AD, indicating that the formation of these tangles is directly linked with neuronal impairment (6). So, the reduction of neurofibrillary tangles can be a therapeutic target for AD treatment (14, 15).

The aggregation of Aβ peptide in extracellular plaque is a neuropathological feature of AD (1, 16). In reality, Aβ peptide, as a principal risk factor in AD, has a major function in beginning and development of this disease (17). Aβ induces oxidative stress, increases reactive oxygen species production and tau hyperphosphorylation, and results in toxic effects on synapses and neurons. For these reasons, treatment with antioxidants such as vitamin E may be helpful in AD (18).

Vitamin E, a potent antioxidant (19), may have beneficial effects in AD on the oxidative stress and Aβ-associated reactive oxygen species production (20). This vitamin may be helpful for the treatment and prevention of AD (21), and its intake can also reduce the risk of AD (22-25). A deficiency of vitamin E is also associated with AD (26), although, the evidence for using vitamin E as a therapeutic in AD is less convincing (21). Epidemiological data show that lower vitamin E levels observed in AD patients are related to a matched control group. In addition, regular supplementation of vitamin E causes the lower rate of development of AD in comparison with the non-supplement users (27). Furthermore, *in vitro* studies indicate a potential role of vitamin E in the prevention of AD (4). However, only one study suggests that vitamin E supplementation with a high dose (2000 IU/d) delays AD progression (28). On the other hand, several contradictory clinical studies indicate that vitamin E supplementation (1000-2000 IU/d) has no benefit in AD patients (29-31) and 400 IU/d of vitamin E supplementation could not prevent dementia (32). Due to the differences in the individual response to vitamin E antioxidant properties, the results on the effect of this vitamin in AD are diverse (33).

Some experiments support the role of vitamin E in alleviating the effects of AD pathology (34). But, the effect of vitamin E as a treatment for AD pathogenesis still needs to be studied. Therefore, we examined the effects of vitamin E treatment on the density of congophilic amyloid plaques and neurofibrillary tangles in the hippocampal subregions of scopolamine-treated rats.

## Materials and Methods

***Animals***

Forty-eight adult male Wistar rats weighing 180–220 g (procured from the Pasteur Institute, Tehran, Iran) were used in this study. The rats were kept one per cage in a temperature-controlled room (22±3 °C) on a 12-hour light/dark cycle with free standard rat pellet food and water. This study was approved by the Ethics Committee at Golestan University of Medical Sciences, Gorgan, Iran.

***Experimental design***

The rats were randomly divided into one control group, two sham groups, and three experimental groups (eight rats per group). The rats in the control group did not receive any drugs. The rats in the sham groups (scopolamine+saline and scopolamine+sesame oil groups) received a single intraperitoneal (IP) injection of scopolamine (3 mg/kg) (35) for one day and then 0.9% saline and/or sesame oil (1 ml/kg, IP) injected for 14 days. The rats in experimental groups (scopolamine+vitamin E group) received a single IP injection of scopolamine for one day and then vitamin E at doses of 25, 50, and 100 mg/kg/day (IP) injected for 14 days (35).

Scopolamine (Tocris, UK) was dissolved in 0.9% saline and vitamin E (Darou Pakhsh Pharmaceutical Co., Iran) in sesame oil.

***Tissue Preparation***

Two days after the last drug injection, the rats were profoundly anesthetized with chloroform and transcardially perfused with 0.9% saline, then with 4% paraformaldehyde (Scharlau, Spain) in phosphate buffer (Gibco, UK). After that, the brains were rapidly collected and fixed in 4% paraformaldehyde for one week. Next, histological processing was performed by an automated tissue processing machine (Did Sabz, Urmia, Iran) and embedded in paraffin. Coronal sections (6 μm) of the hippocampus were prepared with a rotary microtome (Pooyan MK 1110, Iran). The brain sections were stained with Congo red and Bielschowsky staining.

***Congo red staining***

**Figure 1:**
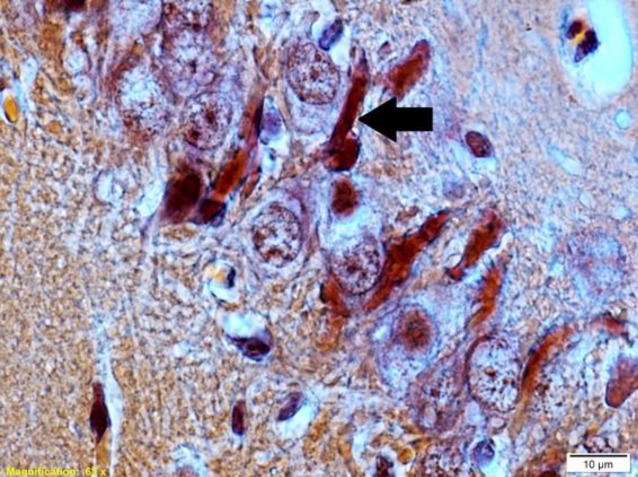
A photomicrograph of Congo red staining in the CA3 region of the hippocampus in the scopolamine+saline group rat. The scale bar is 10 μm. Black arrow show congophilic amyloid plaque in the hippocampal CA3 region

For detection of congophilic amyloid plaque in the hippocampus, brain sections were stained with Congo red (DDK, Italia) (36). Brain sections were first deparaffinized and rehydrated and then dyed with Congo red for 30 min at room temperature. Brain sections were then rinsed in distilled water and alcoholic solution. After this, brain sections were washed in running tap water for 5 min. In the next step, brain sections were counterstained with a Mayer hematoxylin solution for 5 min and then rinsed in running tap water for 10 min. Brain sections were quickly dipped in 95% alcohol (three times) and then dipped twice in 100% alcohol. After that, brain sections were clarified with xylene and finally coverslipped with entellan (Merck, Germany) glue (Figure 1).

***Bielschowsky silver staining***

**Figure 2:**
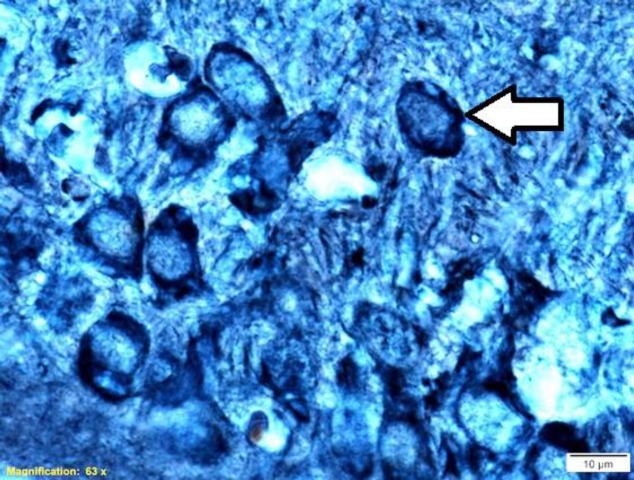
A photomicrograph of Bielschowsky silver staining in CA3 region of the hippocampus in the scopolamine+saline group rat. The scale bar is 10 μm. White arrow show neurofibrillary tangle in the hippocampal CA3 region

Bielschowsky Stain Kit (Shimi Pajohesh Asia, Amol, Iran) was used for staining of the neurofibrillary tangle. After deparaffinization and rehydration, brain sections were washed three times with distilled water. Next, brain sections were placed in 2% silver nitrate solution in the dark at room temperature for 48 hr, followed by washing in distilled water three times; in the next step, ammoniacal silver solution was added to each brain section for 20 min at room temperature and then rinsed three times in distilled water. Afterward, brain sections were reduced in 20% formalin solution for 5 min and washed three times with distilled water. After that, brain sections were placed in 1% gold chloride solution for 60 min at room temperature and rinsed three times in distilled water. Subsequently, 5% sodium thiosulfate solution was added to brain sections for 1 min and washed in running tap water. Finally, the sections were dehydrated through 96% alcohol, absolute alcohol, cleared in xylene, and mounted with entellan glue (Figure 2).

***Image processing and counting***

Images of stained congophilic amyloid plaque and neurofibrillary tangle were captured at 40x magnification by using an Olympus BX53 microscope (Japan) with an attached digital camera (DP73, Olympus, Japan) for the hippocampal CA1 and CA3 and dentate gyrus (DG) regions. Images were routed into a Windows PC for quantitative analyses using cellSens Standard 1.14 software (Olympus, Tokyo, Japan). A field of 12000 μm^2^ was randomly selected for counting the number of neurofibrillary tangles and congophilic amyloid plaque in the hippocampus (12). Imaging and counting were performed blind to the treatment.

***Statistical analysis***

All data were expressed as mean±SD and were analyzed using the one-way ANOVA (SPSS software, version 16.0, Armonk, NY, USA) followed LSD *post-hoc* test. Statistical significance was also set at *P*<0.05.

## Results

***The effect of vitamin E on the number of congophilic amyloid plaques in the hippocampus of male rats treated with scopolamine***

**Figure 3:**
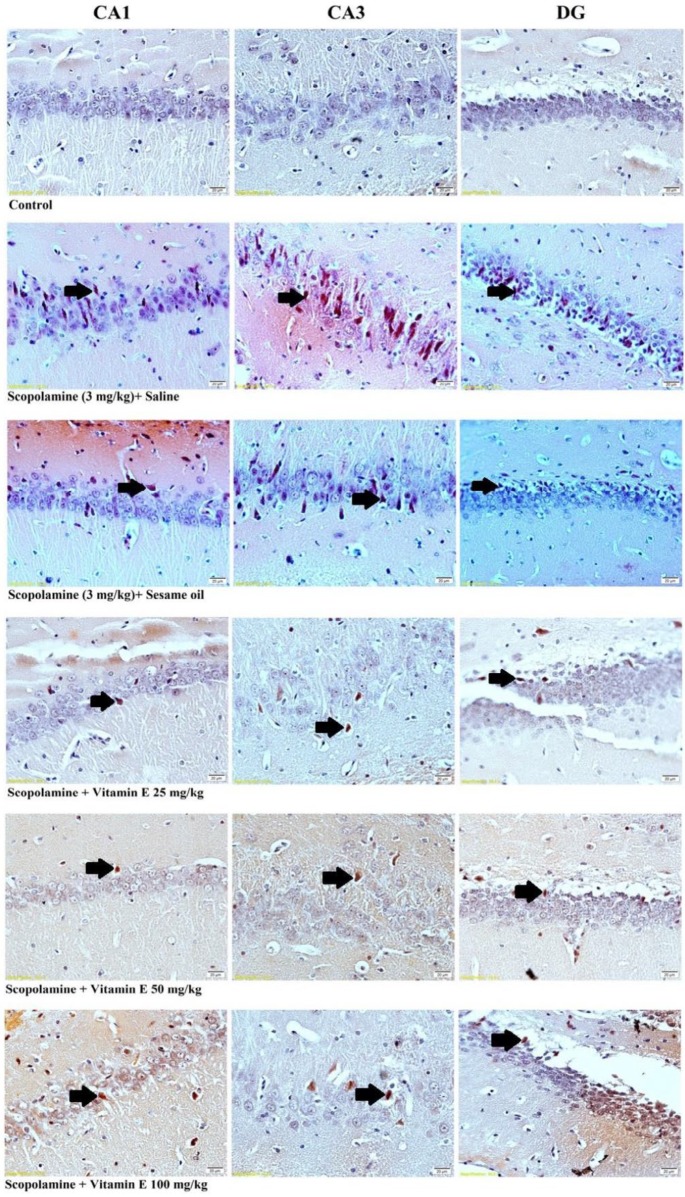
Photomicrographs of Congo red staining in the hippocampal CA1, CA3, and DG regions. Scale bars = 20 μm. Black arrows demarcate congophilic amyloid plaques in the hippocampal CA1, CA3, and DG regions of all groups

In Figure 3, congophilic amyloid plaques are shown in the hippocampal CA1, CA3, and DG areas stained by Congo red staining.

**Figure 4:**
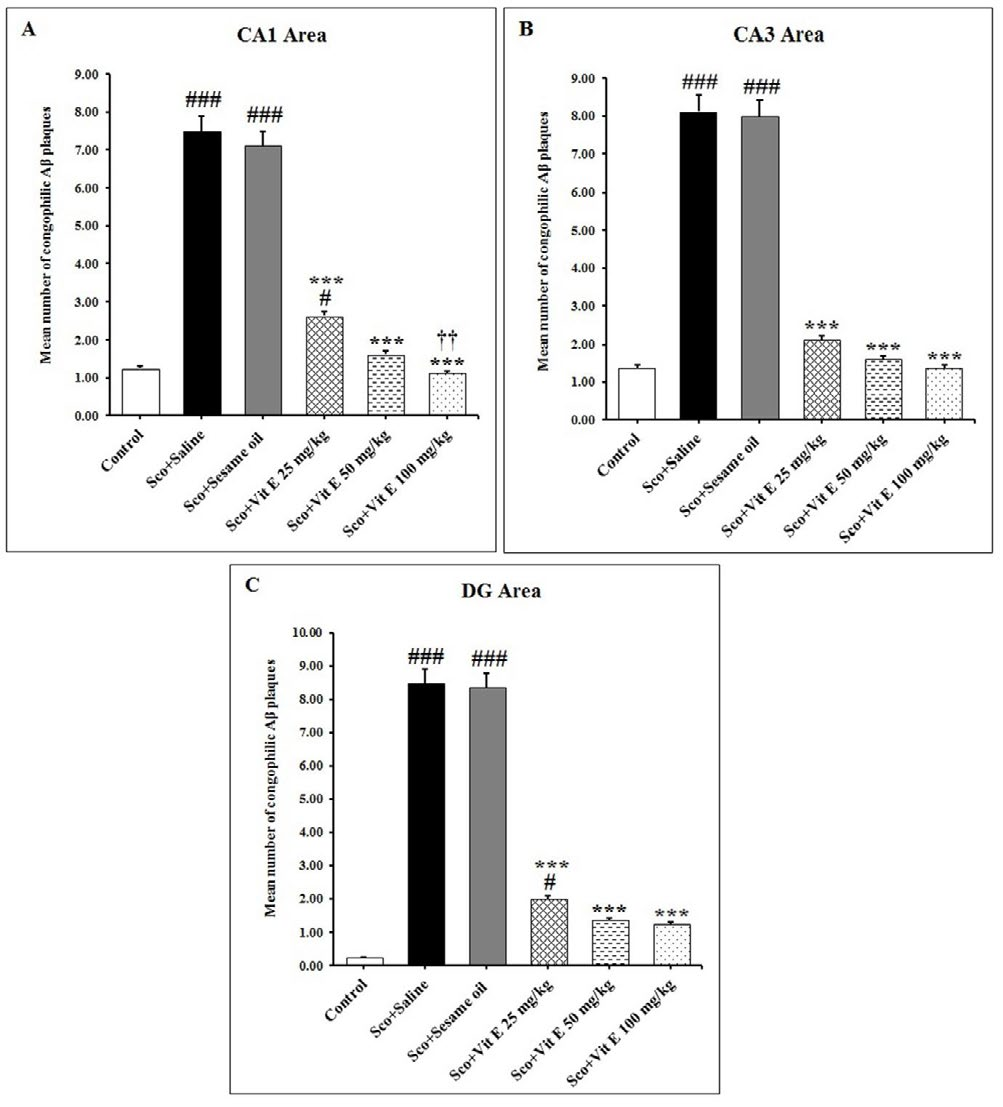
The mean number of congophilic amyloid plaques after administration of vitamin E in the hippocampal CA1 (A), CA3 (B), and DG (C) areas of the of scopolamine-induced AD model rats. Data expressed as mean±SD. # *P*<0.05 and ### *P*<0.001 as compared to the control group; *** *P*<0.001 as compared to the Sco+Saline group; †† *P*<0.01 as compared to the Sco+Vit E 25 mg/kg group. AD: Alzheimer’s disease; Sco: Scopolamine; Vit E: Vitamin E

Figure 4A shows the effects of vitamin E on the congophilic amyloid plaque density in the hippocampus of male rats after the administration of scopolamine. Scopolamine administered rats (sham groups) showed a significant increase in the mean number of congophilic amyloid plaques in the CA1, CA3, and DG regions of the hippocampus as compared with the control group (*P*<0.001, Figures 4A, B, and C). Treatment with vitamin E (25, 50, and 100 mg/kg/day) for 14 days resulted in a significant decrease in the mean number of congophilic amyloid plaques in the CA1, CA3, and DG regions of the hippocampus, compared with the scopolamine+ saline group (*P*<0.001, Figures 4A, B, and C).

Administration of vitamin E showed a dose-dependent effect on the congophilic amyloid plaque density in the rat hippocampus. Moreover, the density of congophilic amyloid plaque in the same regions of the hippocampus was significantly lower in scopolamine+vitamin E 100 mg/kg/day group rats as compared with the scopolamine+ saline group (*P*<0.001, Figures 4A, B, and C). Based on this result, an effective dose of vitamin E was the dose of 100 mg/kg/day. The LSD *post hoc* test revealed a considerable reduction in the congophilic amyloid plaque density in the hippocampal CA1 region after the highest dose of vitamin E (100 mg/kg/day) treatment as compared with the lowest dose of vitamin E (25 mg/kg/day) treatment (*P*<0.01, Figure 4A). However, the congophilic amyloid plaque density showed no significant difference between the vitamin E-treated groups in the CA3 and DG regions of the hippocampus (Figures 4B and C). These data suggest that doses of 25, 50, and 100 mg/kg/day of vitamin E can prevent the scopolamine-induced congophilic amyloid plaque accumulation in the hippocampus.

***The effect of vitamin E on the number of neurofibrillary tangles in the hippocampus of male rats treated with scopolamine***

**Figure 5:**
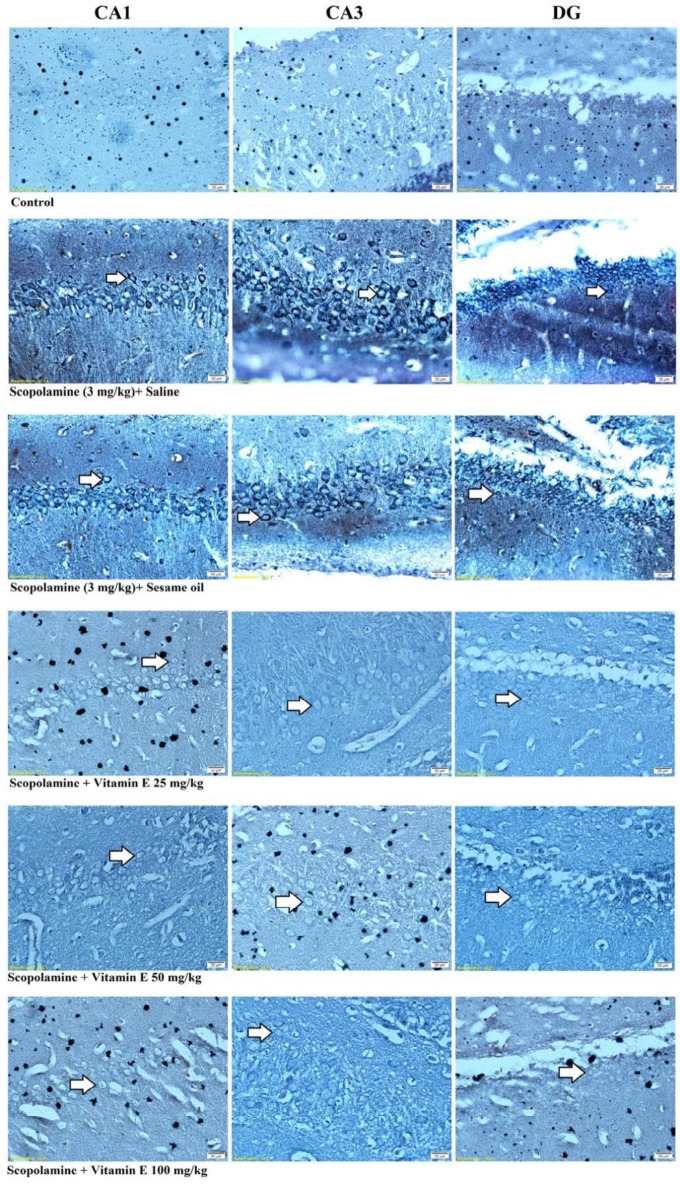
Photomicrographs of Bielschowsky Silver staining in the hippocampal CA1, CA3, and DG regions. Scale bars = 20 μm. White arrows demarcate neurofibrillary tangles in the hippocampal CA1, CA3, and DG regions of all groups

In Figure 5, neurofibrillary tangles were shown in the hippocampal CA1, CA3, and DG areas stained by Bielschowsky silver staining.

**Figure 6:**
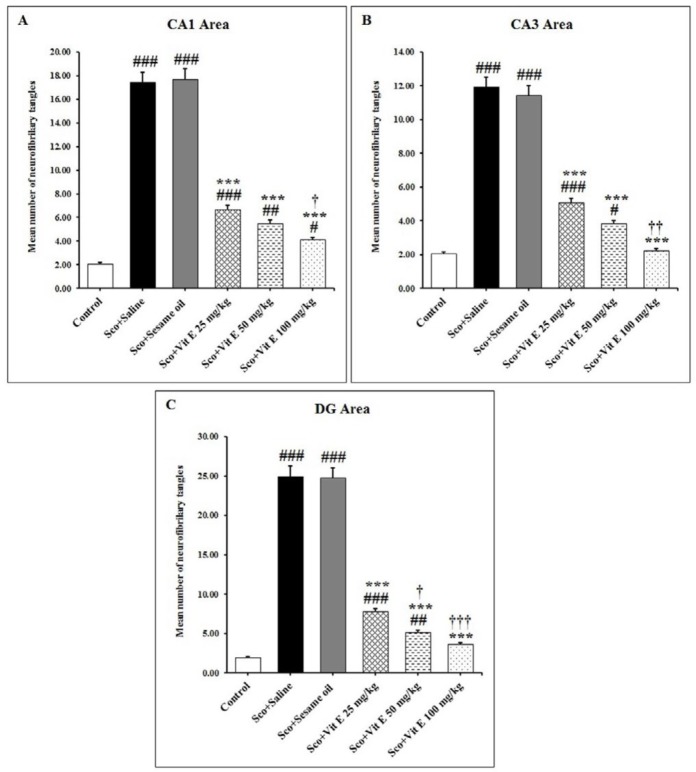
The mean number of neurofibrillary tangles after administration of vitamin E in the hippocampal CA1 (A), CA3 (B), and DG (C) areas of the of scopolamine-induced AD model rats. Data expressed as mean±SD. # *P*<0.05, ## *P*<0.01 and ### *P*<0.001 as compared to the control group; *** P<0.001 as compared to the Sco+Saline group; † *P*<0.05, †† *P*<0.01 and ††† *P*<0.001 as compared to the Sco+Vit E 25 mg/kg group. AD: Alzheimer’s disease; Sco: Scopolamine; Vit E: Vitamin E

The effect of vitamin E on the neurofibrillary tangle density was evaluated using Bielschowsky silver staining in the hippocampus of male rats treated with scopolamine (Figure 6). A single injection of scopolamine significantly increased the mean number of neurofibrillary tangles in the pyramidal layer of CA1 and CA3 regions and the granular layer of the DG region in the sham groups as compared with the control group (*P*<0.001, Figures 6A, B, and C). Vitamin E administration, dose-dependently, significantly decreased the mean number of neurofibrillary tangles in all regions of the hippocampus at 25, 50, and 100 mg/kg/day doses when compared with the scopolamine + saline group (*P*<0.001, Figures 6A, B, and C). This reduction of the neurofibrillary tangle numbers was significantly lower in the pyramidal layer of the CA3 region after treatment with a dose of 100 mg/kg/day of vitamin E compared with the scopolamine+saline group (*P*<0.001, Figure 4B). The LSD *post hoc* test revealed a significant reduction in the neurofibrillary tangle density after high dose vitamin E (100 mg/kg/day) treatment as compared with the control group in the hippocampal CA1 region (*P*<0.05, Figures 6A, B, and C), but not in CA3 and DG regions of hippocampus.

A significant reduction in the mean number of neurofibrillary tangles was also observed in the hippocampal CA1 (*P*<0.05), CA3 (*P*<0.01), and DG (*P*<0.001) regions after treatment with high dose vitamin E (100 mg/kg/day) as compared with the low dose vitamin E (25 mg/kg/day) administration (Figures 6A, B, and C). In rats receiving vitamin E with a 50 mg/kg/day dose a significant decrease in the mean number of neurofibrillary tangles in the DG region of the hippocampus was observed as compared with the low dose of vitamin E-treated rats (*P*<0.05, Figure 6C). Based on this data, a 100 mg/kg/day dose of vitamin E was effective in the reduction of neurofibrillary tangle density. These results indicate that the IP injection of scopolamine increases the neurofibrillary tangle density and that vitamin E treatment for 14 days reduced their density in the hippocampus.

## Discussion

The present study showed that scopolamine-treated rats displayed a higher density of congophilic amyloid plaques and neurofibrillary tangles in the hippocampus. We also observed that congophilic amyloid plaque and neurofibrillary tangle density decreased significantly after 14 days of administration of vitamin E in the hippocampus of AD-like model rats. 

Aβ plaques and neurofibrillary tangles are the two neuropathological symptoms of AD and the accumulation of Aβ in brain areas triggers a pathological cascade in this disease (37). The neurotoxic effects of Aβ in AD are widely studied (11) and these effects include impairing synaptic plasticity, apoptosis, prompting tau phosphorylation, and oxidative stress (10). In this study, we used scopolamine, as an AD-like model that can induce Aβ accumulation and tau hyperphosphorylation in the rodent brain (10-12).

In agreement with our study, the other studies have also found that administration of scopolamine causes an increase in the protein and mRNA levels of the amyloid precursor protein and the Aβ level in the rodent cortex and hippocampus (10, 11, 38, 39). Scopolamine injection also increases the number of congophilic amyloid plaques in the cingulate cortex, cerebellum, hippocampus, and prefrontal cortex of rats (12, 36). They all confirm that scopolamine could induce Aβ accumulation in rodent brains (39).

In some previous studies, scopolamine was found to increase both protein and mRNA levels of tau in rat cortex and hippocampus and also the levels of tau phosphorylated protein in rat hippocampus (11, 38, 40). It is known that the hyperphosphorylation of tau protein and the formation of neurofibrillary tangles are other pathologic symptoms of AD (11). In the current study, we found that a single injection of scopolamine results in an increase of neurofibrillary tangle density in all subregions of the hippocampus.

In addition, the findings of our study demonstrated that treatment with vitamin E decreases congophilic amyloid plaque and neurofibrillary tangle density in the hippocampus of AD-like model rats. As reported before, vitamin E was shown to act against Aβ peptide accumulation in the rodent brain. For example, a diet supplemented with vitamin E reduces Aβ1–40 and Aβ1–42 levels and amyloid deposits in younger transgenic mice model of AD but not in older transgenic mice model of AD (41). Vitamin E treatment (2 IU/g diet, for 4 weeks) can also reduce levels of Aβ1–42 and Aβ burden in the brain of transgenic mice model of AD undergoing repetitive concussive brain injury (42). Additionally, vitamin E can modulate the hippocampal gene expression encoding for proteins required in Aβ elimination, which suggests that vitamin E has a protective effect on the progression of AD (27). It is also reported that feeding animals for six weeks with vitamin E (100 mg/kg) and selenium or their combination with epigallocatechin-3-gallate during AD induction decreased the beta-amyloid content in aluminum chloride-induced rat model of AD (43). One *in vivo *study demonstrated that the administration of vitamin E diets (0.026, 0.04, and 0.2 g/kg, for 7 months) in combination with fish oil supplements can reduce the cortical and hippocampal Aβ plaque burden in transgenic APP/PS1 mice model of AD (44). It is also reported that treatment of the transgenic mouse model of AD with vitamin C and E combination can considerably reduce the Aβ plaque deposition in the hippocampus, but not in the cortex, in comparison with just vitamin C administration (45). In this view, in agreement with earlier studies, the results of our study showed that vitamin E treatment for 14 days causes a significant decrease in the density of congophilic amyloid plaques in the hippocampus of scopolamine-treated rats.

Some studies suggest that vitamin E has a positive effect on tau hyperphosphorylation, the other symptom of AD. Hyperphosphorylated tau can decrease its binding to microtubules and induce tau aggregation, forming neurofibrillary tangles (18). However, little was known about the effect of vitamin E on the density of neurofibrillary tangles, so that the only study showed a positive effect of α-tocopherol on tau pathology. Nakashima et al. indicated that α-tocopherol supplementation can suppress the progression of tau pathology in tau transgenic mice (46). In agreement with this finding, our study showed that 14 days of administration of vitamin E can decrease the density of neurofibrillary tangles in the hippocampus of the AD-like model rats.

On the other hand, some of the experiments showed that vitamin E has no effect on tau pathology. For example, one study with Western blot analysis indicated that vitamin E (0.5 and 1.5 mM) administration for 10 days cannot change the tau protein levels in drosophila. But, vitamin E treatment reverses tau-induced neurodegeneration in drosophila (47). Another study reported that dietary supplementation of vitamin E cannot decrease the phosphorylated tau expression in the brain in a double transgenic mouse model of AD (33). In contrast to these findings, our results demonstrated that IP injection of vitamin E with three doses of 25, 50, and 100 mg/kg/day can decrease the density of neurofibrillary tangles in the hippocampus of scopolamine-treated rats. Furthermore, *in vitro* experiment revealed that the incubation of cultured neurons with beta-amyloid peptide increases the expression of phosphorylated tau, which Trolox can prevent (33). 

It is also known that oxidative stress is the link between Aβ toxicity and tau hyperphosphorylation. Indeed, Aβ increases the oxidative stress that results in phosphorylation of p38 mitogen-activated protein kinase. This kinase uses tau as a substrate and phosphorylates the tau molecules. So mechanistically, Aβ toxicity can be linked to hyperphosphorylation of tau via p38 mitogen-activated protein kinase. In this regard, one study reported that vitamin E can inhibit the activation of p38 mitogen-activated protein kinase, and this is modulated via reduction of oxidative stress. Therefore, vitamin E has a protective effect against the formation of hyperphosphorylated tau (33). Here, we found that vitamin E can prevent the increase in the number of congophilic amyloid plaques and neurofibrillary tangles induced by scopolamine in the rat hippocampus. But, more investigations are needed in order to determine the exact mechanism of action of vitamin E in the reduction of amyloid plaque and neurofibrillary tangle accumulation in the brain tissue of AD model rodents.

## Conclusion

Vitamin E as an antioxidant could exert a therapeutic effect in the reduction of congophilic amyloid plaque and neurofibrillary tangle density in the hippocampus of scopolamine-treated rats, so it could be useful for Alzheimer’s patients.
